# *In Vivo* Wound Healing and Anti-Inflammatory Activities of Leaf Latex of* Aloe megalacantha* Baker (Xanthorrhoeaceae)

**DOI:** 10.1155/2018/5037912

**Published:** 2018-07-02

**Authors:** Leake Gebremeskel, Dayananda Bhoumik, Gereziher Geremedhin Sibhat, Kald Beshir Tuem

**Affiliations:** ^1^Department of Pharmacy, College of Health Sciences, Aksum University, Aksum, Ethiopia; ^2^Department of Pharmacology and Toxicology, School of Pharmacy, College of Health Sciences, Mekelle University, Mekelle, Ethiopia; ^3^Department of Pharmacognosy, School of Pharmacy, College of Health Sciences, Mekelle University, Mekelle, Ethiopia

## Abstract

**Background:**

* Aloe megalacantha* Baker (Xanthorrhoeaceae) is one of the* Aloe* species widely distributed in Ethiopia. The leaf latex of the plant is used for treatment of wounds, inflammation, and other multiple ailments in Ethiopian traditional medicine.

**Purpose:**

The aim of this study was to evaluate* in vivo* wound healing and anti-inflammatory activities of the leaf latex of* Aloe megalacantha* in mice.

**Methods:**

The wound healing activity of the leaf latex of the plant was studied topically by incorporating the latex in simple ointment base in a concentration of 5% (w/w) and 10% (w/w) using excision and incision models. In these models, wound contraction, period of epithelialization, and breaking strength of the wounded skin were determined. Carrageenan induced inflammation of paw model was also used to assess the anti-inflammatory activity of the leaf latex at doses of 200 mg/kg, 400mg/kg, and 600 mg/kg. The level of inflammation suppressions were measured at 1, 2, 3, and 4 hrs after carrageenan injection, and then the percentages of inflammation inhibition were computed as compared with the negative control.

**Result:**

In both wound models, mice treated with 5% (w/w) and 10% (w/w) latex ointment showed a significant (p<0.05) increment in the rate of wound contraction, reduction in epithelialization time, and higher skin breaking strength. Besides, the latex also exhibited a dose-dependent significant (p<0.05) reductions of inflammation as compared to negative control groups.

**Conclusion:**

The overall results of this study demonstrate that the leaf latex of* A. megalacantha* possesses wound healing and anti-inflammatory activities which can scientifically substantiate the traditional use of the plant as a wound healing agent.

## 1. Introduction

Wound can be defined as a disruption in the normal continuity of anatomic structure and functionality of living tissues [1]. The healing of wound is a physiological process by which the body replaces and restores function to damaged tissues [2]. All tissues in the body are capable of healing either by regeneration or by repair mechanisms, through four precisely and highly programmed phases: hemostasis, inflammation, proliferation, and remodeling or maturation phase [3]. Chronic or nonhealing wounds are usual medical problems which represent a significant cause of morbidity, disability, socioeconomic crisis, and mortality for a large portion of the population [4]. Wound management has been challenging because of its multifactorial causes and these numerous factors limit wounds not to heal by single treatment modality. The management of wound in modern therapy involves ensuring adequate debridement, removal of any foreign bodies, pressure off-loading, proper dressings, and assessment and treatment with antibiotics when needed [5]. When acute wound healing does not progress in an orderly and timely manner, or inappropriate treatment of wound occurs, incisions can dehisce; hernias can form; anastomoses can leak; and fistulae can develop [3].

Herbal preparations and their products are considered as an imperative and main source of modern medicine in the globe [6]. As modern health care system alone could not afford the health needs of the entire population of the world, henceforth traditional therapy as well as herbal medicines are the alternative choices for the management of a number of diseases. In Ethiopia, the practice of traditional medicine has stayed in majority of the rural population since long time ago and here one of the plants* Aloe megalacantha* is claimed and commonly used for wound healing purpose which requires scientific validation [7, 8].

*Aloe megalacantha *is a kind of succulent shrub, 0.5–2 m high characterized by erect, ascending or sprawling stems and is distinguished from the related groups occurring in southern, northern, and eastern Ethiopia by its cylindrical-trigonous perianth, 23–30 mm long leaves [9]. The leaf latex of the plant is used topically for the treatment of itches, dandruff, wounds, and systemically for multiple diseases including malaria, diabetes, and ascariasis, whereas the leaf is used for the treatment of ameba, malaria, and abdominal disease, and the root part is also sometimes utilized for the management of impotence and urine retention [7, 8]. Being the traditional use of* A. megalacantha *for treating various wounds and other dermatological problems is evident; its wound healing potential has not been studied and reported yet. Hence, the purpose of the present study was to scientifically verify wound healing effect of the plant and to confirm the traditional claims which may serve as baseline information for further investigation to initiate advanced studies on identification and characterization of the chemical constituent(s) responsible for the wound healing and anti-inflammatory activities.

## 2. Materials and Methods

### 2.1. Plant Material

The leaf latex of* A. megalacantha *was collected from “Wukiro, Genfel kebele, Dongolo” site, which is about 833 kms towards north from Addis Ababa, Ethiopia. The plant was identified by a taxonomist and a voucher specimen (LG001) was deposited at the National Herbarium, College of Natural and Computational Sciences, Addis Ababa University (AAU) for future reference.

### 2.2. Experimental Animals

Healthy adult Swiss albino mice of either sex weighing 25–30 grams and 6–8 weeks of age; and Sprague Dawley rats of either sex weighing about 200–300 grams, aged 3–4 months, were used for this study. The animals were housed in cages 12 hour light/dark cycles [10]. Animals were acclimatized for one week under laboratory condition before the study. They were provided with food pellets and water* ad libitum*. The experiments were conducted in accordance with the internationally accepted laboratory animal use, care, and guideline [11]. For the work proceed, ethical clearance was secured from the Health Research Ethic Committee, Mekelle University (ref no. 1057/2017).

### 2.3. Collection of Leaf Latex

The leaf latex of* A. megalacantha* was collected by cutting the leaves transversally near the base. The latex was then left in open air for 2–4 days to get dried, and it yielded dark brown powder.

### 2.4. Ointment Formulation

Simple ointment of the leaf latex was prepared by using a formula in British Pharmacopoeia described in the Table 1 [12].

### 2.5. Acute Oral Toxicity

Acute oral toxicity study was performed according to the Organization for Economic Co-operation and Development (OECD, 425) and adopted the procedures described by Mulisa et al. (2015) [10, 13]. Five healthy Swiss female mice weighing 23–30 g were acclimatized for five days before the study. In the first day, single mouse was fasted for 3 h, and 2000 mg/kg of the leaf latex was administered. It was observed periodically for 24 hrs for any acute sign of toxicity, then four mice were added and the same dose was administered, and finally further cage side observation for gross behavioral changes was made following the treatment for 14 days.

### 2.6. Acute Dermal Toxicity

The highest concentration of the leaf latex ointment 10% (w/w) was applied thinly and uniformly on shaved back of the rats for a period of 24 hrs. After application of the test substance, cage side observation was performed daily for the next 14 days to notice late development of dermal toxicity [4].

### 2.7. Grouping and Dosing of Animals

In excision model four groups of mice containing six in each were employed. Groups I, II, III, and IV were treated with simple ointment (negative control), 0.2% (w/v) nitrofurazone (positive control), 5% (w/w) latex, and 10% (w/w) latex ointments, respectively. The same grouping and dosing of mice were also used for incision wound model except addition of fifth group for untreated group. In carrageenan induced hind paw edema model, five groups of mice containing six in each were employed. Tween 80 (2%) as a base, indomethacin (10 mg/kg) as a standard, 200 mg/kg, 400 mg/kg, and 600 mg/kg of latex were assigned to groups I-V, respectively. Pilot study was conducted before the commencement of the actual experiment.

### 2.8. Excision Wound Model

The epithelialization period and wound contraction were monitored according to the procedures described by Kokane et al. (2009) and Mulisa et al. (2015) [4, 10].

Wound contraction was evaluated by measuring wound areas on days 3, 5, 7, 9, 11, 13, 15, and 17. Then, the percentage wound closure was calculated to compare wound contraction rates in each wound by using the formula shown below [10].

(1) %  Wound  closure×100%=Wound  area  on first day−Wound  area  on  the specific daywound  area  on  the first day

Falling of scab without leaving raw wound behind was taken as end point of complete epithelialization and the days required for this was measured as period of epithelialization [14].

### 2.9. Incision Wound Model

Breaking strength was measured on the 10th day using continuous water flow technique [15]. Percentage of breaking strength for the latex and reference drug was calculated applying the following formula [16].

(2) Breaking  strength of BS  latex%=BS  latex−BS  S.OBS  S.O×100Breaking  strength of reference %=BS reference−BS  S.OBS  S.O×100Breaking  strength of S.O  %=BSS.O−BS  L.UBS  L.U×100

where S.O is simple ointment; L.U is left untreated

### 2.10. Anti-Inflammatory Activity

The basal volume of the right hind paw of each mouse was determined following 3-4 hrs fasting using digital Plethysmometer (Orchid Scientific and Innovative Pvt., Ltd. India) [17]. Tween 80 (2%) as vehicle, leaf latex of* A. megalacantha *and reference drug was given orally to their respective groups one hour before carrageenan injection. Solution of 0.05 ml, 1% carrageenan in 0.9% saline (w/v) was injected via subplantar into the right hind paw to induce edema. Through the measurement of the volume displaced by the paw, inflammation was quantified 1, 2, 3, and 4 hr after carrageenan injection and was represented as paw volume (ml) variation with respect the basal values [18, 19]. The percentage inhibition of edema for each group was calculated using the following formula described by Owoyele et al. (2009):

(3) $$\begin{array}{c} Percentage\text{ of }inhibition\;\;edema=\frac{Co-Ct}{Co}\times 100 \end{array}$$

where Co is the average inflammation of the control group at a given time; and Ct is the average inflammation of the plant latex or indomethacin treated mice at the same time.

### 2.11. Statistical Analysis

Results of the study were expressed as mean ± SEM. Statistical significance was determined by one-way ANOVA using statistical package for social science (SPSS) version 21.0, followed by Tuckey post hoc test. The data were considered significant at p < 0.05.

## 3. Results

### 3.1. Acute Oral Toxicity Test

There were no overt signs and symptoms of toxicity noticed in the experimental animals in the first 24 hrs. Furthermore, there was no sign of toxicity and mortality noted during the 14 days of cage side observation.

### 3.2. Acute Dermal Toxicity Test

**Figure 1:**
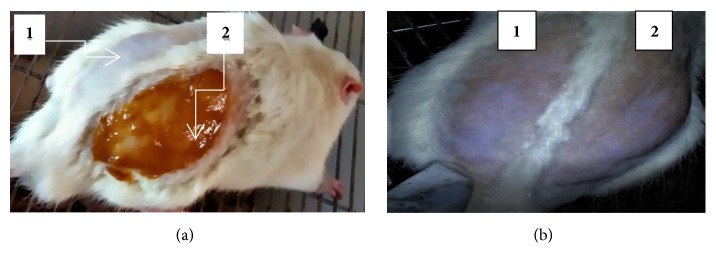
Photographs of acute dermal toxicity test. (a) On the application day, simple ointment (a1) and 10% latex ointment (a2). (b) After 24 hrs application of same rat, simple ointment (b1) and 10% latex ointment (b2).

**Figure 2:**
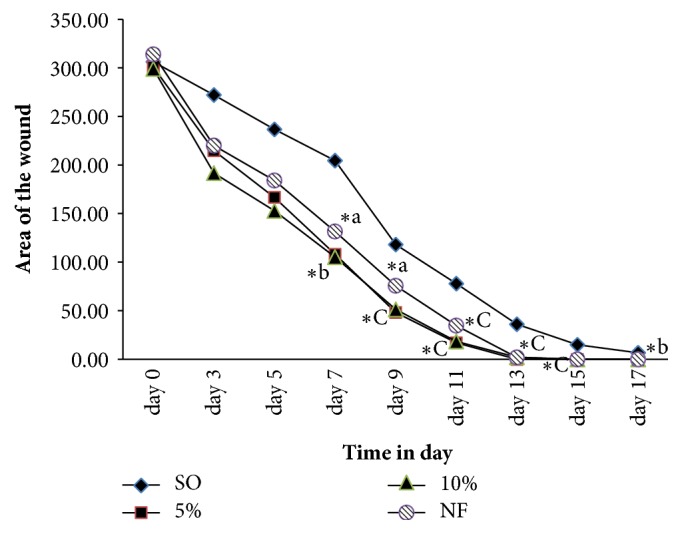
Effect of topical application of leaf latex of* A. megalacantha *Baker on wound contraction in excision wound model in mice. n = 6 Swiss albino mice in each group; values are expressed as mean ± SEM, one-way ANOVA, SO: simple ointment, AML:* A. megalacantha *latex, NF: bitrofurazone, ^*∗*^ against negative control, ^a^p< 0.05, ^b^p< 0.01,^c^p<0.001.

After 24 hrs application of the maximum concentration of the latex ointment (10% w/w), the site did not show any sign of inflammation or irritation (Figure 1, (2b)). No sign of toxicity were observed when the rats were followed for 48 hrs. Moreover, there was no any sign of toxicity detection during the 14 days side cage observation.

### 3.3. Excision Wound Model

#### 3.3.1. Measurement of Wound Contraction

As described in Figure 2, enhanced wound contraction was observed on both dose levels of the latex and the reference drug from 7th up to 13th post wounding days. Both 5% (w/w) and 10% (w/w) of the latex ointments showed higher wound closure than standard drug in all days of application though no apparent difference in activity was detected.

**Figure 3:**
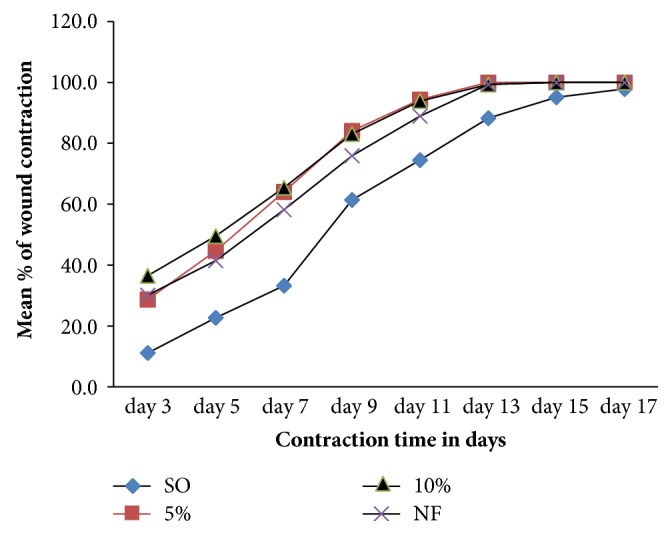
Effects of the leaf latex of* A. megalacantha* on wound closure of excision wound model.

In the 5% (w/w) leaf latex treated mice, the maximum rate of wound contraction was observed on 11th (94.4%), 13th (100%), and 15th (100%) days after wound creation. This rate of wound closure was comparable with (11th (88.58%), 13th (99.42%), and 15th (100%)) of nitrofurazone treatment days (Figure 3).

#### 3.3.2. Epithelialization Time Measurement

The leaf latex of* A. megalacantha* was found to reduce the time required for epithelialization of the excised wound in mice. Prominent significant (p<0.001) reduction of epithelialization time was observed in animals treated with 5% (w/w), 10% (w/w) latex, and 0.2% w/v nitrofurazone ointments as compared to simple ointment treated control group (Table 2). The 5% (w/w) latex showed little shorter time in epithelialization period than both 10% (w/w) latex and reference drug treated animals.

### 3.4. Incision Wounds

#### 3.4.1. Breaking Strength

As shown in Table 3, the leaf latex of* A. megalacantha* was effective in increasing the breaking strength of the healed wound. Enormously significant (p<0.001) difference in breaking strength was observed in all groups treated with 5% (w/w), 10% (w/w) latex, and 0.2% w/v nitrofurazone ointment.

### 3.5. Anti-Inflammatory Test

**Figure 4:**
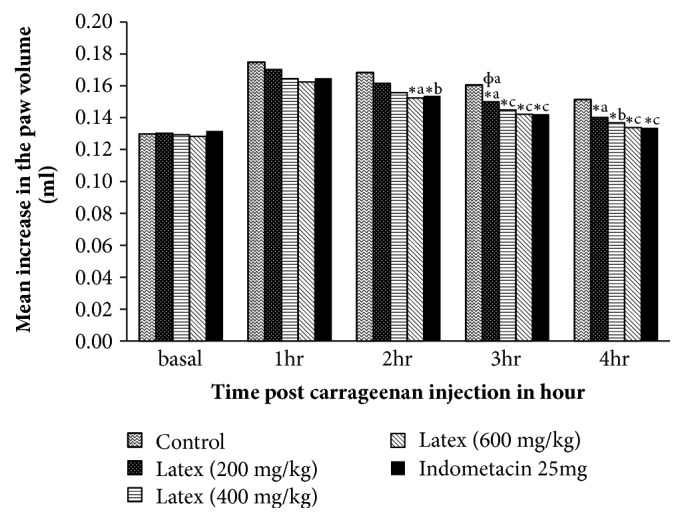
Anti-inflammatory activity of the leaf latex of* A. megalacantha* in carrageenan induced mice paw edema model. Each value represents mean± SEM, ^*∗*^as compared to negative control, ^*ɸ*^as compared to 200 mg/kg of the latex, ^a^p< 0.05, ^b^p<0.01, ^c^p< 0.001, Values in parenthesis represent percentage inhibition of paw volume.

Significant reduction of paw edema began with 600 mg/kg latex (p< 0.05) and indomethacin (p< 0.01) administered groups following 2 hrs carrageenan administrations as compared to control mice (Figure 4).

**Figure 5:**
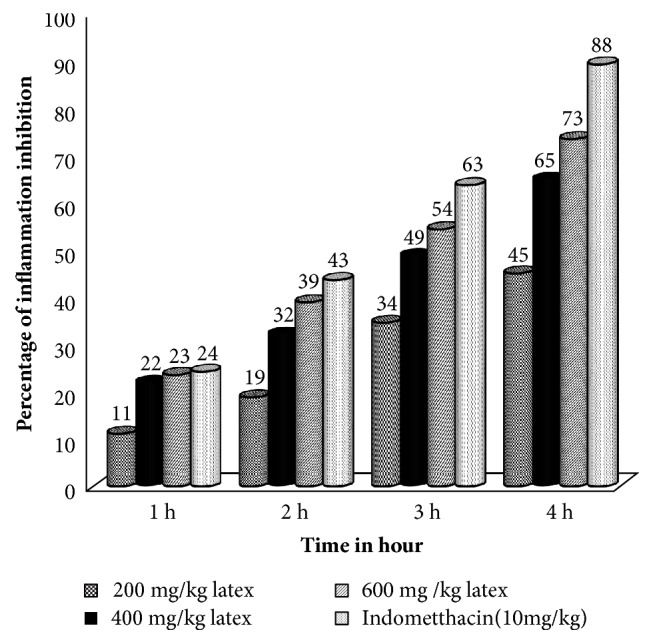
Percentage inhibition of carrageenan induced paw edema by* A. megalacantha* and indomethacin in mice.

Significant reduction in inflammation was observed with middle (400mg/kg) (p<0.01) and the higher (600 mg/kg) doses of the latex (p<0.001) as compared to the negative control group at 3hr post-carrageenan administration (Figure 4). The percent inhibition of edema was a bit higher in indomethacin than all dose levels of the latex in all time course of the study, but failed to reach statistical significance level (Figure 5).

## 4. Discussion

Since wound healing is a complex cascade, it is difficult to reveal all healing processes using single model or* in vitro* experiment [20]. In this study, results obtained from excision and incision wound models indicated that the effect of both 10% (w/w) and 5% (w/w) latex ointment treated animals showed significant enhancement of wound healing as compared to controls. As described in Figure 2, 5 % (w/w) latex showed a minute higher in healing activity than 10 % (w/w) latex though the difference in activity was not significant. This could be suggested that irritant compounds in the latex may cause recurrent inflammation, imped healing, on the wounded site after multiple application of higher dose 10% (w/w) during the course of the study. Similar findings were also reported from previous studies [21, 22]. Wound healing effect of both latex ointments of* A. megalacantha* was little higher than the standard drug (nitrofurazone). The reason might be individual or multiple of phytochemical constituent(s), present in the latex of* A. megalacantha,* could better facilitate wound healing possibly by acting through multiple mechanisms than the standard which was consistent with similar results described in previous study [10].

Rate of wound contraction and the epithelialization period were used as variables in excision wound healing. Wound contraction, part of the proliferative phase, is the centripetal movement of the edges of a full thickness wound and occurs throughout the healing process and it possesses 88% of the healing wound and the rest 12% is added by scar formation [23].

Starting from 7th postwounding day onwards, the mice treated with both dose levels of the latex showed significant wound contraction as compared to control group. On 13th day of postwound induction, the excised wounds treated with 5% (w/w), 10% (w/w) latex ointment, and standard drug were significantly closed by 100%, 99.27%, and 99.42% respectively; and the epithelialization date was very significantly reduced from 18 days to 12, 12.33, and 13 days respectively once again. In contrast, simple ointment (the negative control) treated animals contracted 88.26% of the excised wound on same day of measurement about five days later, which is in line with Beshir et al., (2016). Higher anti-inflammatory effect or induction of macrophage cell proliferation, stimulation of fibroblast migration from the wound edge to the wound site, and proliferation and production of collagen (main component of extracellular matrix) could be the possible mechanisms of the latex for its wound healing potential [24].

The breaking strength was measured in incision wound model as a parameter which shows how much the repaired tissue resists to breaking under tension. The percentage of increase in breaking strength of animal wounds treated by 5% (w/w), 10% (w/w) latex, and 0.2% nitrofurazone ointments were 77.81%, 74.47%, and 71.78% respectively. However, the percentage of the breaking strength of simple ointment treated mice was 23.88%, which was threefold less than the percentage of 5% (w/w) latex ointment. The effect could be due to the presence of secondary metabolites responsible for enhancement of collagen maturation which gives strength and integrity to the wound matrix. Furthermore, the increment in tensile strength may be associated with the promotion of collagen synthesis, angiogenesis, and stabilization of fibers and hence the overall effect improves circulation for oxygen and nutrients supply that are vital for wound healing cascade [25].

The anti-inflammatory activity of the leaf latex of the plant was another contributing factor for wound healing effect. Inflammation is a defense mechanism and self-limiting when it is regulated physiologically. During acute wound, it prepares the wound bed to be healed by removing dead cells, debris and bacterial contaminates as well as recruiting and activating fibroblasts that are necessary for production of collagens and ground substances. However, uncontrolled and excessive inflammation limits wound healing which increase the level of matrix metalloproteinase (MMPs) that can degrade the extracellular matrix (ECM) and ends up with delayed wound repair processes [26].

Carrageenan induced paw edema model has been used widely for the discovery and evaluation of anti-inflammatory drugs [17]. Three distinct phases appeared after carrageenan injection: Histamine and serotonin mediators involved in the first phase (0-1.5h); bradykinin involvement in the second phase (1.5-2.5 h) and followed by the third phase (2.5-5 h) in which production of large amount of proinflammatory mediators such as prostaglandins (PGE_2_) and various proinflammatory cytokines such as interleukin-1 beta (IL-1 *β*), interleukin-6 (IL-6), and tumor necrosis factor alpha (TNF *α*); and infiltration of neutrophils into the inflammatory site take place. The third phase is responsive to most anti-inflammatory drugs; and hence it is frequently used to investigate the antiedematous effect of natural products [18].

Anti-inflammatory effect of the latex was noted at 2, 3, and 4 hrs post-carrageenan injection in a dose-dependent manner. Two hours after carrageenan injection, only the higher dose of the latex (600 mg/kg) and indomethacin administered mice showed significant inhibition of the edema. It could be due to lower doses (200 mg/kg, 400 mg/kg) of the latex might not be able to achieve maximum plasma concentration at 2 hr for second phase edema inhibition. After 4 hr (late phase) carrageenan injection; 200 mg/kg, 400 mg/kg, 600mg/kg) doses of the latex and indomethacin inhibited the edema by 44.84%, 64.78%, 73.11% and 88.23% respectively. Thus, it is plausible to say that the latex could contain phytochemical constituents potent and effective in inhibiting the release and/or the activity of bradykinin and/or prostaglandins in the third phase of edema formation [18]. Other suggested mechanisms for the higher suppression of edema could be inhibition of cyclooxygenase [27].

## 5. Conclusion

Results obtained from the present study indicated that the leaf latex of* A. megalacantha* increased wound contraction and breaking strength of the repaired tissue and reduced the time for epithelialization. Furthermore, the plant latex was also endowed with anti-inflammatory activity which can explain partly for its wound healing effect. These findings highlight the potential wound healing activity of the plant's latex and justify its traditional claims for treatment of wounds.

## Figures and Tables

**Table 1:** Master and reduced formula used for simple ointment preparation.

Ingredients	M.F (grams)	R.F ( grams)
Wool fat	50	10
Hard paraffin	50	10
White soft paraffin	850	170
Cetostearyl alcohol	50	10
Qs	1000	200

M.F = master formula; R.F = reduced formula.

**Table 2:** Effect of topical application of leaf latex of *A. megalacantha* on epithelialization period of excision wound model in mice.

Groups	Period of epithelialization (days)
Simple ointment	17.83±0.307
0.2% w/v Nitrofurazone	13.00±0.258_ _^*∗*c^
5% w/w latex	12.00±0.516_ _^*∗*c^
10% w/w latex	12.33±0.715_ _^*∗*c^

n = 6, Swiss albino mice in each group; values are expressed as mean ± SEM, one-way ANOVA. *∗* as compared to negative control, ^c^p<0.001.

**Table 3:** Effect of topical application of the leaf latex of *A. megalacantha* on breaking strength in incision wound model.

Groups	Breaking strength (gram)	% Breaking strength
Untreated control	173.17±6.253	_
Simple ointment	214.00±7.095	23.88
0.2% w/v Nitrofurazone	365.50±11.955_ _^*∗ɸ*c^	71.48
5% w/w latex	378.83±14.809_ _^*∗ɸ*c^	77.81
10% w/w latex	372.83±12.111_ _^*∗ɸ*c^	74.47

n = 6, Swiss albino mice per group, values represents mean ± SEM, ^*∗*^ as compared to simple ointment, ^*ɸ*^ as compared to untreated control. ^c^p< 0.001.

## Data Availability

The data used to support the findings of this study are available from the corresponding author upon request.

## Abbreviations

ANOVA: Analysis of variance
BP: British pharmacopoeia
ECM: Extracellular matrix
OECD: Organization for economic cooperation and development
SEM: Standard error of the mean.
